# A novel QoS-aware prediction approach for dynamic web services

**DOI:** 10.1371/journal.pone.0202669

**Published:** 2018-08-22

**Authors:** Yiguang Song, Li Hu, Ming Yu

**Affiliations:** 1 School of Electronic and Info rmation Engineering, Hebei University of Technology, Tianjin, China; 2 National Space Science Center, Chinese Academy of Sciences, Beijing, China; 3 School of Software Engineer, Beihang University, Beijing, China; Beijing University of Posts and Telecommunications, CHINA

## Abstract

Web service has become irreplaceable for service-oriented application in both academia and industry in recent years. Quality of Service (QoS) is used to describe the nonfunctional characteristics of Web service. Identifying Web service QoS is crucial for service-oriented application designers because service users may obtain very different QoS performance of the same service in the client-side due to dynamic changes of Internet environment as well as user context. However, evaluating QoS performance of a large scale of Web services requires considerable time and resources in real-world. Existing methods can make a personalized prediction for average QoS values by employing historical data but fail to take into consideration the fluctuation feature of Web service QoS values. To address this issue, this paper proposes a novel method for personalized QoS prediction of dynamic Web Services. First, a novel approach is used to extract feature points of QoS sequences and dynamic time warping distance is used to compute the similarity instead of Euclidean distance. By finding the most similar QoS sequences of the target QoS sequence, the missing QoS values can be predicted without extra Web services invoking. To validate our method, we conduct a large number of experiments based on real-world Web service QoS data set. The experimental studies show that our method has higher accuracy rate compared with the existing methods.

## Introduction

Web services are self-described applications designed to support interoperable machine-to-machine interaction over the network via standard interfaces and communication protocols [1]. Quality of Service (QoS) is usually employed to describe the nonfunctional Characteristics of Web service [2]. With the increasing number of Web service and extensive application in various domains, there are many Web services providing by different service providers with the same or similar functionalities but difficult QoS values. Thus, how to identify the QoS of Web service with identical or similar functionalities is a fundamental problem in service computing.

Generally, service providers usually provide an official QoS value of all their services. However, different service users invoking the same service from the same service provider may observe various QoS performance because the network environment changes over time and over user context. To obtain the local QoS values of the target service, service users usually send a request of target service in client-side and wait for the result from server-client. However, it’s difficult for service users to conduct all the real-world Web service measurements at the client-side and sometimes it’s impossible due to the following reasons:

- First, Web service invocations may consume a large amount of time and cost a lot of money because many Web service is charged. Although some providers could provide free services, it still takes a long time.
- Second, it's time-consuming for service users to evaluate Web service. Most service users are not professional in evaluating services. And some services are difficult to measure and need long-term observation and detection.
- Finally, some evaluation in the client-side is unreal for other service users considering the different context and dynamic network environment.

Therefore, providing a time-aware QoS prediction method is urgent for service-oriented application designers to make decisions of Web service selection [3] [4], Web service composition [5] [6]. Existing approaches can only predict the average QoS values but fail to predict the fluctuation of QoS of dynamic Web service.

We make a full analysis of factors affecting prediction accuracy. First, due to sparse active users, top-k similar users may include negatively similar users in the process of similarity calculation. Second, some dissimilar services may be included in the prediction process and cause a negative influence on predicted results. Active users may invoke only a few Web services and some approaches make a prediction based on a lot of web service invoking. Third, many of their methods employ the average QoS value of an active user to describe the historical data. However, the average performance of Web services will lead to finding dissimilar users or services of the active user because QoS values vary from network to network and location to location.

To solve the problem above, we propose a novel method for personalized QoS prediction of dynamic Web services. The idea is utilizing the QoS values of user usage experience to make a prediction of the QoS value of requested service. To get a higher similarity of the current user and current service, we extract the RTT fluctuation feature of all services and employ the feature matrix to compute the similarity of services and users to find the most similar services and most similar users. Then the missing values are predicted by the weighted calculation of historical data.

In summary, this paper makes four obvious contributions as follows:

- Provide a prediction model for dynamic Web services QoS values, which support all the metrics of QoS such as respond time and throughput without any modifications.
- Propose a method to extract feature points of QoS time sequences to reduce calculation time greatly especially when the dataset is very large.
- The first work to explicit dynamic time warping distance instead of Euclidean distance to measure the similarity of Web service QoS time sequences.
- Predict the personalized QoS values of Web services with a high accuracy, and support real-time service selection and service composition.

The remainder of this paper is organized as follows: section II presents the background of QoS prediction. Section III describes our method and shows our method how to make a prediction of the missing values. Section IV shows the experiment and the results. Section V introduces the related work. Section VI makes a conclusion on this paper.

## Materials and methods

Web service becomes an important part of service-oriented applications and QoS is the key factor for their designers to choose a trustworthy web service from many candidate services. Besides service recommendation also need QoS performance data of all users and service.

Section III-A describes the problem of QoS value prediction on dynamic web services and then we solve the problem in five phrases. First, we extract feature points of dynamic web service time sequences in Section III-B and then Section III-C shows how to calculate the distance between two QoS sequences. In Section III-D and Section III-E, we compute the similar users and similar web services of the target sequence based on the dynamic time warping distance. Finally, a prediction is made in Section III-F based on previous methods.

### Problem description

The aim of our method is to predict the missing QoS values in a high accuracy so that service user can select an optimal QoS web service in candidate services. The process of prediction is always involving a user-service matrix, as shown in Table 1.

**Table 1:** User-service matrix.

	Service1	Service2	Service3	Service4	Service5
**User A**	***q***_**1,1**_	***q***_**1,2**_	***q***_**1,3**_	NAN	***q***_**1,5**_
**User B**	***q***_**2,1**_	***q***_**2,2**_	***q***_**2,3**_	NAN	***q***_**2,5**_
**User C**	***q***_**3,1**_	NAN	NAN	***q***_**3,4**_	NAN
**User D**	***q***_**4,1**_	***q***_**4,2**_	***q***_**4,3**_	NAN	***q***_**4,5**_
**User E**	NAN	***q***_**5,2**_	***q***_**5,3**_	***q***_**5,4**_	***q***_**5,5**_

Table 1 shows a toy example of the user-service matrix. Here are five users and five services in the table. And each entry is a QoS value vector of one web service observed by one service user.

Table 1 shows a toy example of the user-service matrix. Here are five users and five services totally. In this matrix, every entry is a QoS value vector (e.g. *q*_1,1_ to *q*_5,5_) of one property (e.g. respond-time, availability, price, reliability.) of one web service (e.g. service1 to service5) observed by one service user (e.g. user *A* to user *E*). For example, user *A* invokes web service1 a few times and the vector of these respond-time values will be recorded as the first entry. The *NAN* value in the matrix means the service user has not invoked the service yet and has no record about QoS of the service. Since service users invoke a few web services in the real world, the user-service matrix is very sparse.

The problem is how to employ the existing entries in predicting the *NAN* entries in the user-service matrix.

More formally, the problem studied in this paper is defined as follows: Given a set of users *U* and a set of services *S* as well as a set of vectors *Q* of QoS values (In this paper, we mainly focus on the property of respond-time, which also called Round-trip time (RTT)), predict the missing values in the user-service matrix using the existing QoS values provided by different users.

Here is the definition of notations used in the rest of this paper:

***U*** = {*u*_*i*_|1 ≤ *i* ≤ *n*} is a collection of service users, where *n* is the total number of service users and *u*_*i*_ stands for a service user in this collection.

***S*** = {*s*_*j*_|1 ≤ *j* ≤ *m*} is a collection of web services with similar functionality, where *m* is the total number of web services and *s*_j_ stands for a web service in this collection.

***Q*** = {*q*_*i*,*j*_|1 ≤ *i* ≤ *n*, 1 ≤ *j* ≤ *m*} is a collection of QoS vectors and each element is a QoS values vector of a web service observed by a service user. For example, user B observes several QoS values of web service1 and these values is noted as *q*_2,1_.

### Problem definition

given a sparse user-service-time three-dimensional matrix *M*, we will use the existing entries *q*_*i*,*j*_ to predict the missing values.

### Method description

First, input the target user *uid* and target service *sid*. Second, extract feature points *FP* of all the time sequences *q*_*i*,*j*_, and then find the similar users and similar services of the target sequence via computing the dynamic time warping distance of *FP*. Finally, predict the target sequence with the similar sequences.

The details in our method will be introduced in the following five phases.

### Related work

To improve the prediction accuracy, many researchers have proposed a series of QoS predicting methods, generally including the two categories: memory-based methods, model-based methods. The details will be introduced in corresponding sections.

#### Memory-based methods

The idea of memory-based methods is employing the similarity between web services and between service users to predict the target QoS values. Shao et al. [7] firstly proposed Collaborative Filtering (CF) to predict QoS values of unused web services. This method assumes that web service users with the similar experience on some services will have similar experiences on the other web services. It calculates the similarity between every two users and predicts the unused web service QoS values based on the users with a higher similarity of the target user.

Lin et al. [8] improved the CF method and proposed to measure the similarity between web services with Euclidean distance. This method gives a QoS prediction method considering both user similarity and web service similarity. However, the prediction accuracy is undesirable by only employing the similar user's experience. In order to improve the prediction accuracy, Zheng et al. [9] proposed a prediction method based web service historical information. This method firstly calculates the similarity between the target service and other services and selects top-k similar services to fill in the missing values of QoS information matrix. Then it predicts the target according to the QoS information matrix and further improves the prediction accuracy.

Most of the existing methods are based on a large number of web services QoS data. However, there is a lack of relatively complete dataset for most researchers, which makes it hard to do further research. Zhang et al. [10] put forward a WSPred framework, which is a collaborative platform to share QoS information among users and facilitate the system to make accurate prediction and recommendation. This method implements the user-side and lightweight middleware to realize the information recording and QoS experience sharing. This paper predicts the missing QoS value by analyzing the user characteristics, the time characteristics, and the service characteristics.

#### Model-based methods

The model-based approach is an improved prediction method based on the CF method. There are three main models including cluster models [11][12][13], matrix decomposition models[14][15] and tensor decomposition models[16][17]. Clustering models focus on grouping users and services according to different clustering ways, and then calculating similarity based on them, so as to calculate the target QoS values.

Since the traditional prediction methods cannot identify user characteristics, Chen et al. [11] designed a novel cluster-based prediction method and put forward the concept of regional sensitive services and regional sensitive users. This paper predicts according to the regional sensitive users and sensitive services. Compared with the traditional collaborative filtering algorithms, this method achieves higher accuracy and can predict the personalized QoS values of a target object.

Yu et al. [12] proposed a novel algorithm based on time and geographical location to predict QoS value. This algorithm solves three major problems of recommendation system: high-quality prediction, high maintainability, easy modeling, and maintenance. Without traversing the entire QoS dataset, this method only needs to find similar services and users. However, when the number of users increases or the number of services increases, only partial clustering results in inaccurate prediction. How to improve the processing capability of high dimensional datasets is a challenge.

To overcome the shortcomings of the existing CF methods, Liu et al. [13] proposed a location-aware QoS prediction method considering the geographic location of users and web services in order to enhance the reliability of the similarity. Geographic location information of the service and the user is fully taken into account, and a more personalized recommendation is made for the user. In fact, as far as response time is concerned, the geographically similar web services do perform better than far geographically located web services.

Compared with other CF methods, this method predicts closer to the original QoS values in the real world. However, it does not consider the dynamic change in the web environment.

The above two kinds of prediction method based on memory and model can improve the prediction accuracy to some extent, but they cannot predict the dynamic fluctuation because of the dynamic web environment. Usually, the QoS of web service is strongly related to the web server's load, network transmission rate, task type and task size. One or more of these factors will cause the change of web service QoS. Even the same service invoking by the same user will be observed different QoS values at different time slices. These QoS prediction methods are unable to predict the sequence of the target user and target service. Our method fills the blank of these previous methods and makes the first step to predict the QoS sequence.

### Phase1: Extracting feature points

Usually, we use the average QoS value to replace a QoS value vector for the convenience of computing. However, the average values cannot deliver the characteristics of dynamic web services and may lead to finding dissimilar services or service users.

In fact, there are a set of key points in a time sequence. These key points can characterize the original sequence. So, we can extract these points to shorten the original sequence while the dataset is very large. We can save a lot of time via computing feature sequences instead of original time sequences.

**Definition 1**: if a point *q*_*i*_ in sequence S = {*q*_1_,*q*_2_,…,*q*_*n*_} meets the following condition (1), *q*_*i*_ is a feature point.

$$\left\{q_{i}|VD(q_{i})=\max\;\left(VD(q_{j})\right),j=1,2,\ldots ,n\right\}$$ (1)

**Definition 2**: VD is the vertical distance from points A (*i*, q_*i*_) and line B. Line B is the line connecting start points and end point. VD is defined as follows:

$$VD=q_{i}‑(a\times i+b)$$ (2)

Notes: linear equation of Line B is defined as *Y* = a*X*+b. Given two points *M* and *N*, we can get the Coefficient *a* and *b* by solving the linear equation.

We first connect start point *P*_1_ and *P*_*n*_ and get a line *l*. Then via computing *VD* of all points, we get one feature point *P*_*i*_ of sequence *S*. *P*_*i*_ divides *S* into two subsequences. Then we adopt the same steps as mentioned above, we will get all the feature points until the length of all subsequences is less than stop_s (stop_s is decided by users). The stop_s means compress original time sequences into a short sequence with 1/stop_s length.

**The algorithm 1**: extracting feature points of dynamic web service time sequences

**Input**: a sequence of QoS values s = {q_1_,q_2_,…,q_n_}, stop_s

**Output**: a feature sequence of QoS values *newS*

1: startPoint := 1

2: endpoint := n

3: calculate Coefficient *a* and *b* of the line connect startPoint and endPoint

4: for i := 1 to n

5:         VD[i] = q_i_-(a*i+b)

6: end

7: middlePoint := max(VD)

8: newS.push(middlePoint)

9: left := middlePoint—startPoint

10: right := endPoint—middlePoint

11: if(left > stop_s)

13:         *newS* = getFP(startPoint, middlePoint, *s*)

14: if(right < stop_s)

15:         *newS* = getFP(middlePoint, endPoint, *s*)

16: if(left < = stop_s & right > = stop_s)

17: **return** *newS*

### Phase2: Dynamic time warping distance

After extracting the feature points of time sequences, we get feature sequences of original. But how to measure the similarity of two temporal sequences is the first problem to solve.

In time sequences analysis, Dynamic Time Warping (DTW) is the classical algorithm for computing the similarity between two given sequences. Instead of Euclidean distance, DTW distance is employed for only one reason: DTW can measure the distance of two sequences which may not be aligned in time. Traditional Euclidean distance only computing the similarity of two time-aligned sequences.

The algorithm of DTW is described as follows. We use the idea of dynamic programming to solve the problem.

**Algorithm 2**: DTW Distance Computing

**Input**: two sequences of QoS values s_1, s_2

**Output**: DTW distance dt

1: DTW := array [0..n, 0..m]

2: for i := 1 to n

3:         DTW[i, 0] := infinity

4: for j := 1 to m

5:         DTW[0, j] := infinity

6: DTW[0, 0] := 0

7: for i := 1 to n

8:         for j := 1 to m

9:             cost: = d(s_1[i], s_2[j])

10:                 DTW[i, j] := cost + min(DTW[i-1, j ],

11:                     DTW[i, j-1],

13:                     DTW[i-1, j-1])

14: dt = DTW[n, m]

15: return dt

### User-based prediction

Given a user set *U* and a service set *S*, the target user *u* invokes a set of service *S*_*u*_. we use the following equation (3) to the get the most similar user *best*_*u* of target user *u*. ∈

$$best\_u=\min_{i\in U,\;q,t\in S}\;\left(DTW(S_{u,q},S_{i,t})\right)$$ (3)

*S*_*u*,*q*_ is the sequence of service *q* observed by target user in different time slices and *S*_*i*,*t*_ is the sequence of services *t* observed by user *i* in different time slices. *DTW*(*S*_*u*,*q*_,*S*_*i*,*t*_) is the dynamic time warping distance between the sequence *S*_*u*,*q*_ and the sequence *S*_*i*,*t*_. The *best*_*u* is the user id of minimum DTW distance in all the sequences.

For high prediction accuracy, we use the following equation (4) get the similarity coefficient of the *best*_*u* and the target user *u*.

$$Coef(best\_u,\;u)=\frac{\Sigma_{i\in S_{uv}}S_{u,i}}{\Sigma_{j\in S_{uv}}S_{\mathrm{bes}t_{u},j}}$$ (4)

*S*_*u*,*i*_ is the sequence of service *i* invoking by target user and *S*_*best*_*u*,*j*_ is the sequence of service *j* invoking by the most similar user *best_u*. Service *i* and *j* are selected from *S*_*uv*_, which is defined in equation (5).

$${S_{uv}=S}_{u}\cap S_{v}$$ (5)

*S*_*u*_ is the service collection invoking by user *u* and *S*_*v*_ is the service collection invoking by user *v*. *S*_*uv*_ is the service collection of common service both invoking by the target user and the best user.

Based on the previous steps, a user-based prediction can be made in (6), where *S(best*_*u*,*s)* is the sequence of service *s* invoking by the most similar user of all the users.

Prediction_S(u,s)=Coef(best_u,u)×S(best_u,s) (6)

### Service-based prediction

Given a user set *U* and a service set *S*, the target user *u* invokes a set of service *S*_*u*_. We use the following equation (7) to the get the most similar user *best*_*s* of target user *s*.

$$best\_s=\min_{i\in S,\;q,t\in U}\;\left(DTW(S_{q,s},S_{t,i})\right)$$ (7)

*S*_*q*,*s*_ is the sequence of target service *s* observed by the user *q* in different time slices and *S*_*t*,*i*_ is the sequence of services *i* observed by user *t* in different time slices. *DTW*(*S*_*q*,*s*_, *S*_*t*,*i*_) is the dynamic time warping distance between the sequence *S*_*q*,*s*_ and the sequence *S*_*t*,*i*_. The *best*_*s* is service id of minimum DTW distance of all the sequences.

For high prediction accuracy, we use the following equation (8) get the similarity coefficient of the service *best*_*s* and the target service *s*.

$$\mathrm{Coef}(best\_s,\;s)=\frac{\sum_{m\in U_{\mathrm{ij}}}S_{m,s}}{\sum_{n\in U_{ij}}S_{n,best\_s}}$$ (8)

S_m,s_ is the sequence of target service *s* invoking by user *n* and *S*_*n*,*best*_*s*_ is the sequence of the most similar service *best*_*s* invoking by user *m*. User *n* and *m* are selected from *U*_*ij*_, which is defined in equation (9).

$$U_{ij}=U_{i}\cap U_{j}$$ (9)

*U*_*i*_ is the user collection which invokes service *s* and *U*_*j*_ is the user collection which invokes service *best*_*s*. *U*_*ij*_ is the common user collection which both invokes the target service *s*.

Based on the previous steps, a service-based prediction can be made in (10), where S(*u*,*best*_*s*) is the sequence of best service *best*_*s* invoking by the target user.

Prediction_U(u,s)=Coef(u,best_s)×S(u,best_s) (10)

### Hybrid Prediction

Based on the user-based prediction and service-based prediction, hybrid prediction in (11) weights *λ* for user-based prediction and weights 1 − *λ* for service-based prediction.

P=λ×Prediction_U+(1−λ)×Prediction_S (11)

## Results and discussion

QoS prediction is a highly common problem of web service recommendation and we conduct a lot of experiments to validate the accuracy of our prediction method compared with other classical approaches such as item-based Pearson Correlation Coefficient (IPCC) [18] and user-based Pearson Correlation Coefficient (UPCC) [19]. According to our experiments, we address these following issues:

- How does our method compare with other prediction methods? There are so many prediction methods in recent literature and a few can predict the fluctuation of QoS sequences. which method can be used to compare with our proposal?
- How does data density affect the prediction accuracy of our method? Data density usually is used to test prediction method accuracy in order to simulate the true scenario in real-world. In fact, service users only invoke a few web services but in experiments, a service user always invokes thousands of web services. So we randomly remove some records to get the density of 5% to 50%.
- How does ***λ*** affect prediction accuracy of our method? In the hybrid prediction method, it gives different weights on the most similar service and the most similar user. We do a lot of experiments to figure out which affects the prediction accuracy more.

Section A describes details about the dataset and Section B introduces the evaluation metrics. Section C shows the experimental procedure and outcomes of five prediction methods. And Section D and Section E explain the influence of matrix density and weight. Finally, Section F gives a discussion about the results.

### Data set description

We adopt the dataset released by [20], which contains about 150 million Web service invocation records of 4,532 Web services from 142 users at 64-time intervals. Every time interval takes 15 minutes. RTT records are collected by 150 computer nodes from Plant-Lab, which are distributed in more than 20 countries.

To evaluate the performance of these algorithms above, in reality, we randomly remove RTT records from the original data to generate a sparse training data and compare the predicted values with original ones. Because only a small number of Web service would be used by users in reality.

### Evaluation metrics

Mean Absolute Error (MAE) and Root Mean Squared Error (RMSE) is a statistical accuracy metric and commonly used in collaborative filtering to measure the prediction accuracy. We assess the prediction quality of our proposal compared with other approaches by computing MAE and RMSE. For all test users and test services, MAE is defined as follows:

$$\mathrm{MAE}=\frac{\sum_{uid,\;sid}\left|{\hat{S}}_{uid,\;sid}‑S_{\mathrm{uid},\;\mathrm{sid}}\right|}{L}$$ (12)

RMSE is defined as follows:

$$RMSE=\sqrt{\frac{\sum_{uid,sid}{\left({\hat{S}}_{uid,sid}-S_{uid,sid}\right)}^{2}}{L}}$$ (13)

Where ${\hat{S}}_{\left(uid,\;sid\right)}$ denotes the predicting temporal sequence of user *uid* and service *sid*, *S*_*uid*,*sid*_ represents the time sequence of service *sid* observed by the user *uid*, *L* denotes the length of the predicting temporal sequence.

### Comparison

We conduct a lot of experiments to evaluate our approach. We implement the method as well as baseline approaches using the Matlab code and pack them into a web service. So, readers who are interested in our method can invoke the web service at any time. All the experiments were conducted on a PC with Intel i7-3537U CPU and 8G DDR3 RAM, running 64-bit window 10 operating system. All the source code (include Matlab and python code) will be released on my personal website and well documented. In order to evaluate the accuracy of our method, we compare the accuracy of our method with a baseline approach and other two QoS prediction methods. Although these methods cannot predict the fluctuation of dynamic web service QoS values directly, we can use the improved methods to predict.

- **M1 (UMEAN)**: this method predicts the target QoS values by employing the user average QoS values observed by the other users of the target user.
- **M2 (IMEAN)**: this method predicts the target QoS values by employing the service average QoS values observed by the other services of target service.
- **FFM-1**: this method is our proposed method, Fluctuation Feature Method-1, predicting QoS sequences based on users by Eq (5). It extracts the feature points of the time sequences and predicts QoS values via calculating the DTW distance and finds the most similar users with the target user.
- **FFM-2**: this method is our proposed method, Fluctuation Feature Method-2, predicting QoS sequences based on services by Eq (9). It extracts the feature points of the time sequences and predicts QoS values via calculating the DTW distance and finds the most similar services with the target service.
- **HY**: this method is our proposed method, the Hybrid prediction method combined user prediction and service prediction, predicting QoS sequences by Eq (10). It makes a prediction by employing the most similar services and users of the target object and giving them different weights.

In order to demonstrate the effectiveness of our method, we compare the MAE and RMSE values of respond time and throughput respectively with the above methods. We compare each prediction results with original data and calculate the values of MAE and MRSE. A lower numerical value of MAE or MRSE is considered better performance. In a real-world scenario, users always invoke only a few web services and a lot of web service are unused. So we remove some entries randomly and get a matrix with a density of different percent(e.g., 5, 10, 15 and 20 percent). For example, a 10 percent matrix means we select 10 percent data from the user-service matrix and use the selecting data to predict the remaining 90 percent data. Since the IMEAN and UMEAN method cannot predict the QoS sequence directly, we extend them into M1 and M2 to predict.

In Table 2, we compare the performance of these five methods above in the same parameter setting. We set λ = 0.8, and density = 5% to 5%, step = 5% in this experiment. As we can see in Table 2, the HY method has small values of both MAE and RMSE in the respond-time dataset from density = 5% to 20% and improve 68.9%~77.5% than M1. From density = 25% to 40%, FFM-1 are observed lower values of MAE and RMSE. And from density = 45% to 45%, HY method has lower values of MAE and RMSE again.

**Table 2:** Performance comparison.

Matrix Density	Metrics	Respond Time(seconds)					Throughput(kbps)				
		M1	M2	FFM-1	FFM-2	HY	M1	M2	FFM-1	FFM-2	HY
5%	MAE	0.5666	0.5465	0.4259	0.6020	**0.1759**	11.1014	10.9140	**8.4673**	13.4059	9.4127
	RMSE	0.5668	0.5467	0.4482	0.6091	**0.6645**	11.8297	11.6540	**9.3238**	14.0696	10.2470
10%	MAE	0.5480	0.5277	0.1406	0.5731	**0.1232**	11.0448	10.3358	**6.9002**	11.4556	7.7327
	RMSE	0.5482	0.5279	0.1455	0.5837	**0.4231**	11.7766	11.1136	**7.7827**	12.1665	8.6106
15%	MAE	0.5351	0.5008	0.1128	0.5847	**0.0669**	10.9026	10.0073	**7.4370**	11.2546	8.1416
	RMSE	0.5353	0.5010	0.1230	0.5849	**0.0834**	11.6433	10.7990	**8.3172**	11.9734	9.0177
20%	MAE	0.5160	0.4787	0.1012	0.6214	**0.0892**	10.7245	9.3053	**5.5049**	11.0461	6.4318
	RMSE	0.5162	0.4789	0.1096	0.6217	**0.0906**	11.4767	10.1320	**6.3260**	11.7753	7.2871
25%	MAE	0.5029	0.4525	**0.0698**	0.6151	0.1125	10.6779	8.9408	**5.7583**	11.2191	6.6909
	RMSE	0.5032	0.4528	**0.0751**	0.6154	0.1136	11.4333	9.7831	**6.5958**	11.9410	7.5560
30%	MAE	0.4825	0.4196	**0.0815**	0.6151	0.0842	10.1845	8.2679	**5.5914**	11.2890	6.5517
	RMSE	0.4827	0.4199	**0.0862**	0.6154	0.0859	10.9685	9.1164	**6.4196**	12.0058	7.4114
35%	MAE	0.4714	0.3933	**0.0635**	0.6095	0.0768	10.0723	7.6662	**5.5387**	19.5954	6.5484
	RMSE	0.4717	0.3936	**0.0692**	0.6100	0.0786	10.8612	8.5134	**6.7155**	20.1242	7.7452
40%	MAE	0.4471	0.3693	**0.0708**	0.6074	0.0748	9.3885	7.1600	**4.4101**	9.3053	4.9439
	RMSE	0.4473	0.3696	**0.0761**	0.6078	0.0767	10.2106	8.0039	**5.1943**	10.0941	5.8228
45%	MAE	0.4192	0.3405	0.0853	0.6136	**0.0653**	9.2562	6.8917	**5.8972**	7.7779	6.2457
	RMSE	0.4195	0.3408	0.0898	0.6142	**0.0675**	10.0857	7.7322	**6.7358**	8.6310	7.0570
50%	MAE	0.4141	0.3208	0.0847	0.6512	**0.0683**	9.0017	6.5425	**4.4567**	10.4833	5.1425
	RMSE	0.4144	0.3212	0.0893	0.6514	**0.0701**	9.8421	7.3646	**5.2532**	11.1860	6.0468

Table 2 shows the performance of each method we compared in the same parameter setting. We compared the MAE and RMSE of five prediction methods changing matrix density from 5% to 50% on two datasets. Each entry is the value of MAE or RMSE observed by the experiments.

In the throughput dataset, we observed FFM-1 has smaller MAE and RMSE values than the other four methods from density = 5% to 50%. These methods are observed different performance with different matrix density. In this experiment, HY has a better performance than the other methods in general.

### Influence of matrix density

**Fig 1:**
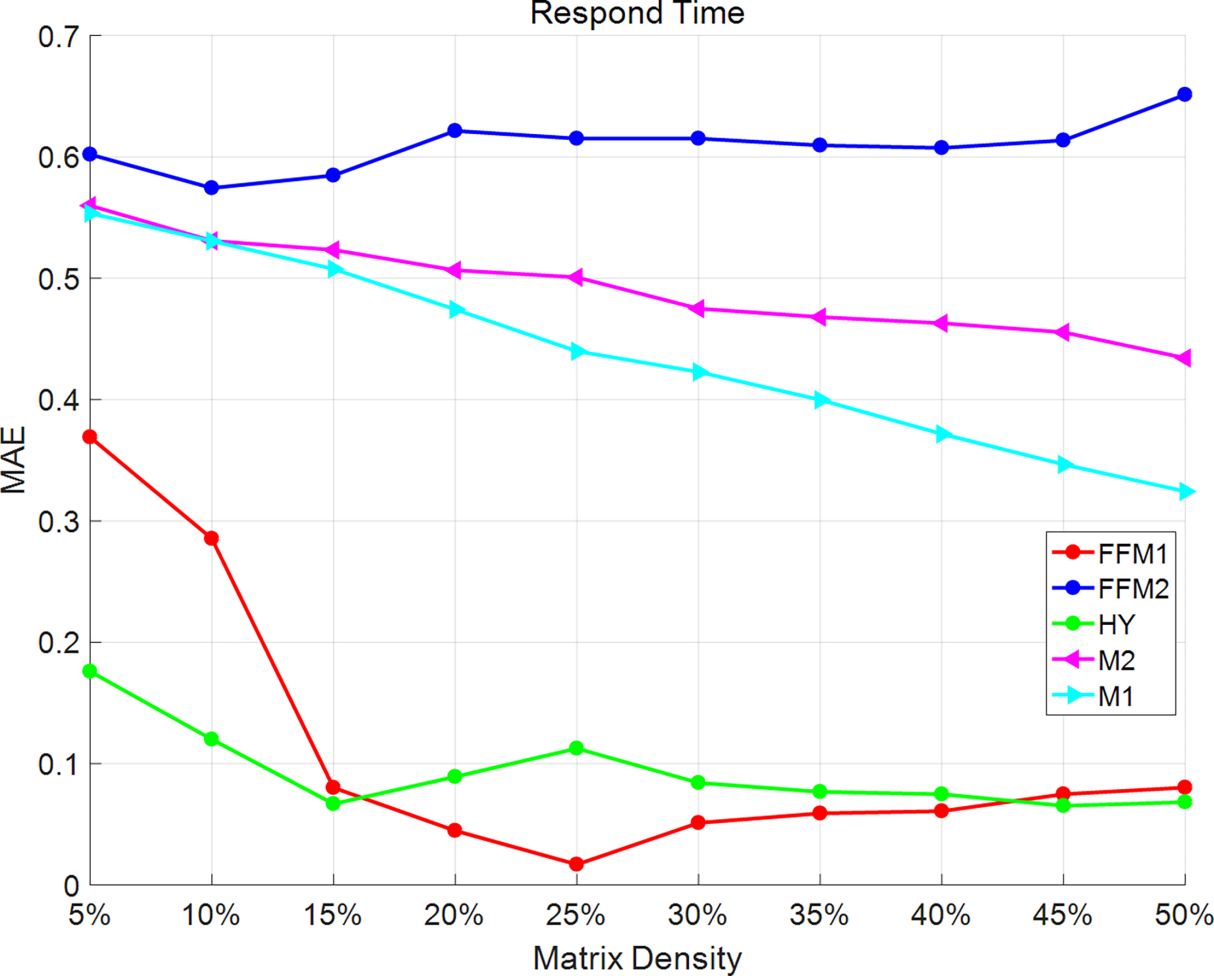
MAE changes with matrix density on the respond-time dataset. In the respond-time dataset, MAE of M1 and M2 methods get smaller as the matrix density increases. MAE of FFM2 methods have no obvious changes with matrix density. In FFM-1 and HY methods, the overall trends of MAE become smaller as matrix density changes.

**Fig 2:**
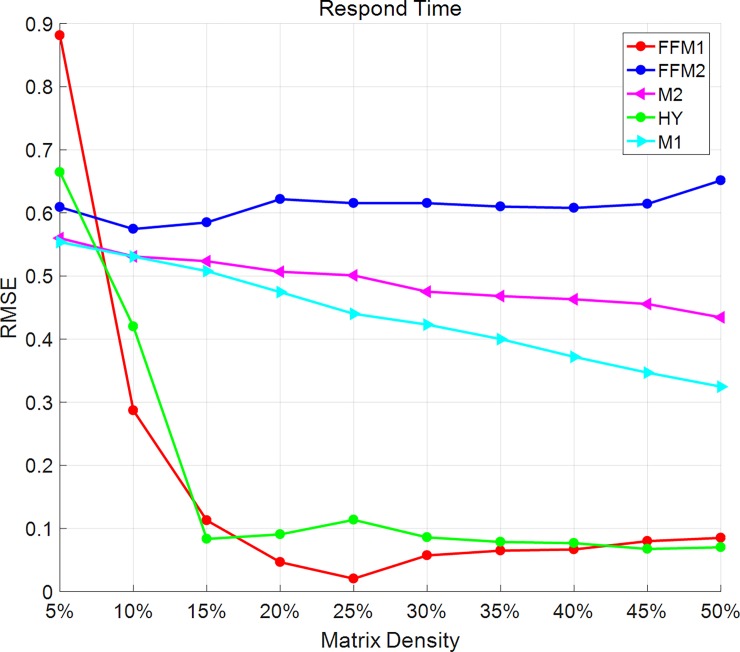
RMSE changes with matrix density on the respond-time dataset. In the respond-time dataset, RMSE of M1 and M2 methods get smaller as the matrix density increases. RMSE of FFM2 methods have no obvious changes with matrix density. In FFM-1 and HY methods, the overall trends of RMSE become smaller as matrix density changes.

To further evaluate the influence of matrix density, we compare all the predict accuracy changing density from 5% to 50% with a step of 5%. As Figs 1 and 2 shows, in the respond-time dataset, MAE and RMSE of M1 and M2 methods get smaller as the matrix density increases. However, in the FFM2 method, MAE and RMSE have no obvious changes with the matrix density. In FFM-1 and HY method, the overall trends of MAE and RMSE are becoming smaller as the matrix density changes. 15% and 25% are two turning points for HY methods. From 5% to 15% and 45% to 50%, HY method has smaller values of MAE and RMSE than FFM-1 method. From 20% to 40%, HY method has bigger values of MAE and RMSE than FFM-1 than FFM-1.

**Fig 3:**
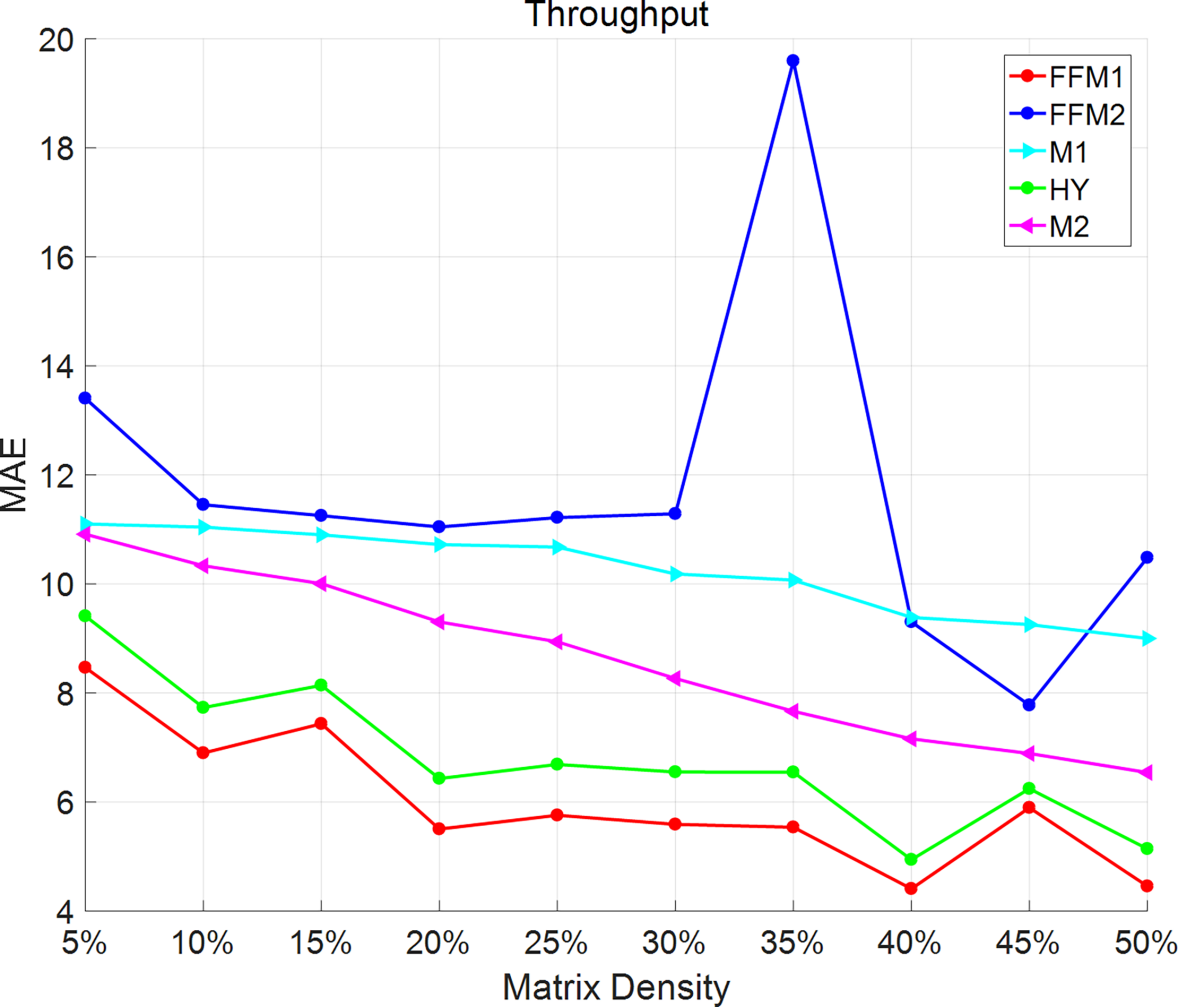
MAE changes with matrix density on throughput dataset. In throughput dataset, all the methods except FFM-2 have a downward trend of MAE.

**Fig 4:**
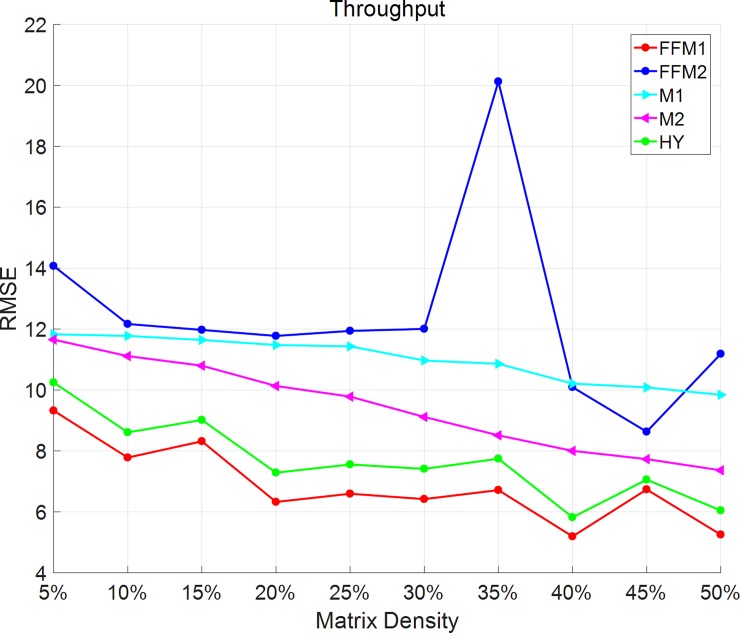
RMSE changes with matrix density on throughput dataset. In throughput dataset, all the methods except FFM-2 have a downward trend of RMSE.

As Figs 3 and 4 shows, in throughput dataset, all the methods except FFM-2 have a downward trend of MAE and RMSE. When matrix density varies from 5% to 50%, FFM-1 has small values of MAE and RMSE than the other methods. FFM-2 has an overall decreasing trend except for the point of 35%. Due to the difference between datasets, these methods may perform a little different. In generally speaking, MY method and FFM1 has a non-controversial advantage than the other methods.

### Influence of *λ*

From the experiment of matrix density on the respond-time dataset, 15% and 25% are two turning points for HY methods. We do a lot of experiments to study the influence of weight in two datasets. We set the same density in the same experiment to get the values of MAE and RMSE using the prediction method HY.

**Fig 5:**
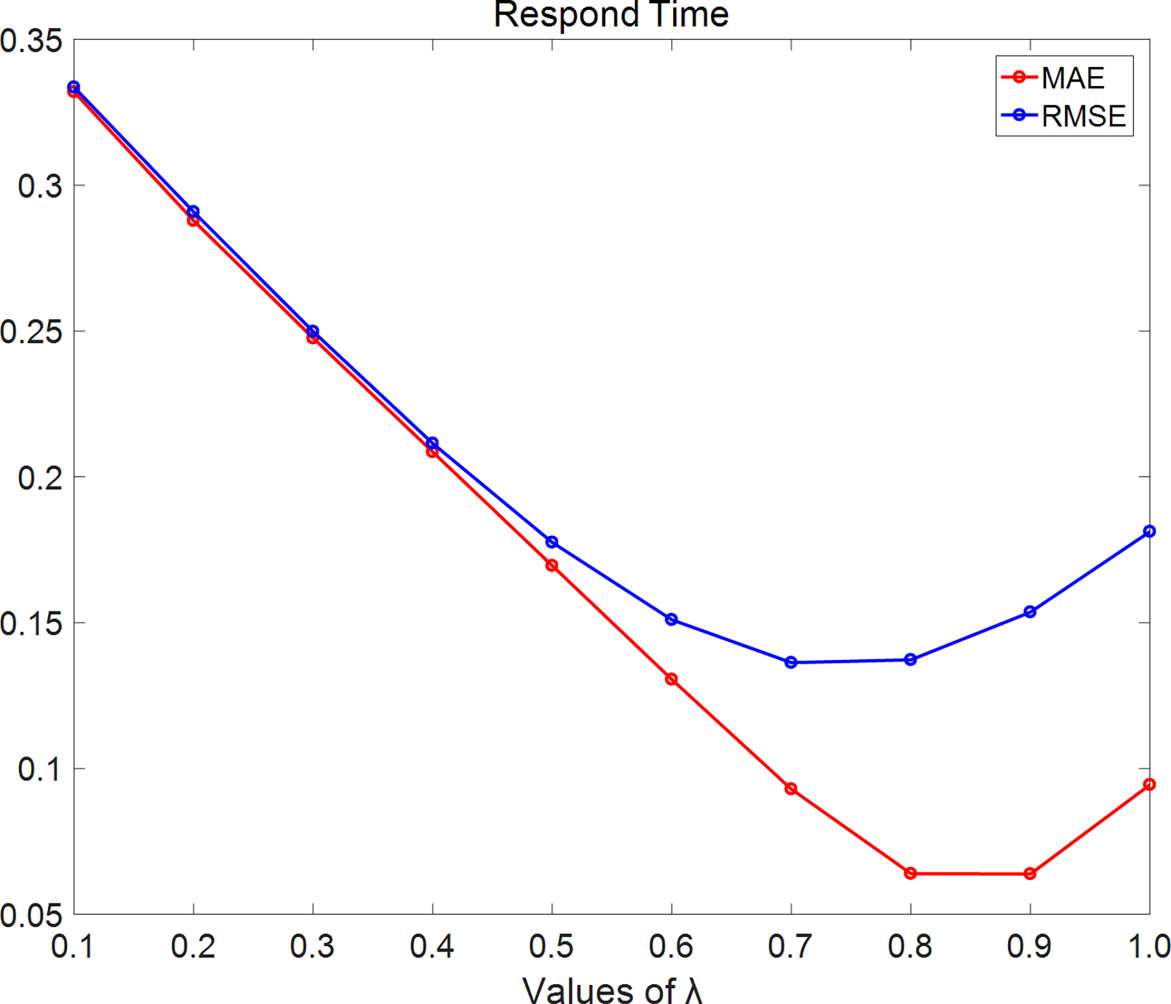
MAE changes with λ on the respond-time dataset. In this experiment, we set density = 10% and using respond-time dataset. As the figure shows, MAE and RMSE firstly decrease with λ while λ<0.8 and then increase with λ while λ≥0.8.

In Fig 5, we set density = 10% and using respond-time dataset. As the figure shows, MAE and RMSE firstly decrease with λ while λ< 0.8 and then increase with λ while λ≥0.8.

**Fig 6:**
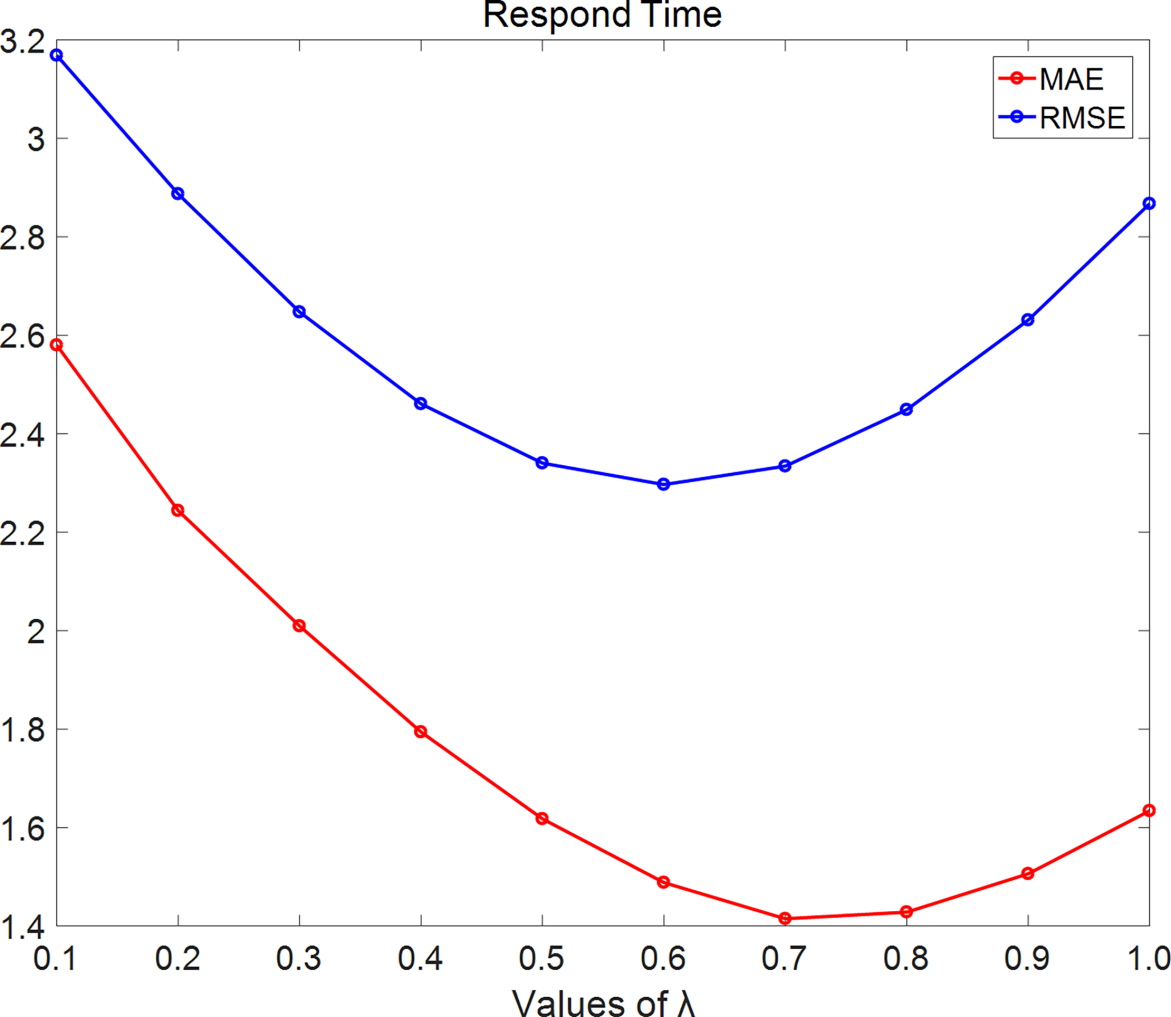
RMSE changes with λ on the respond-time dataset. In this experiment, we set density = 25% and using respond-time dataset. As the figure shows, MAE firstly decreases with λ while λ<0.7 and then increases with λ while λ≥0.7. RMSE decreases with λ while λ<0.6 and then increases with λ while λ≥0.6.

In Fig 6, we set density = 25% and using respond-time dataset. As the figure shows, MAE firstly decreases with λ while λ< 0.7 and then increases with λ while λ≥0.7. RMSE decreases with λ while λ< 0.6 and then increases with λ while λ≥0.6.

**Fig 7:**
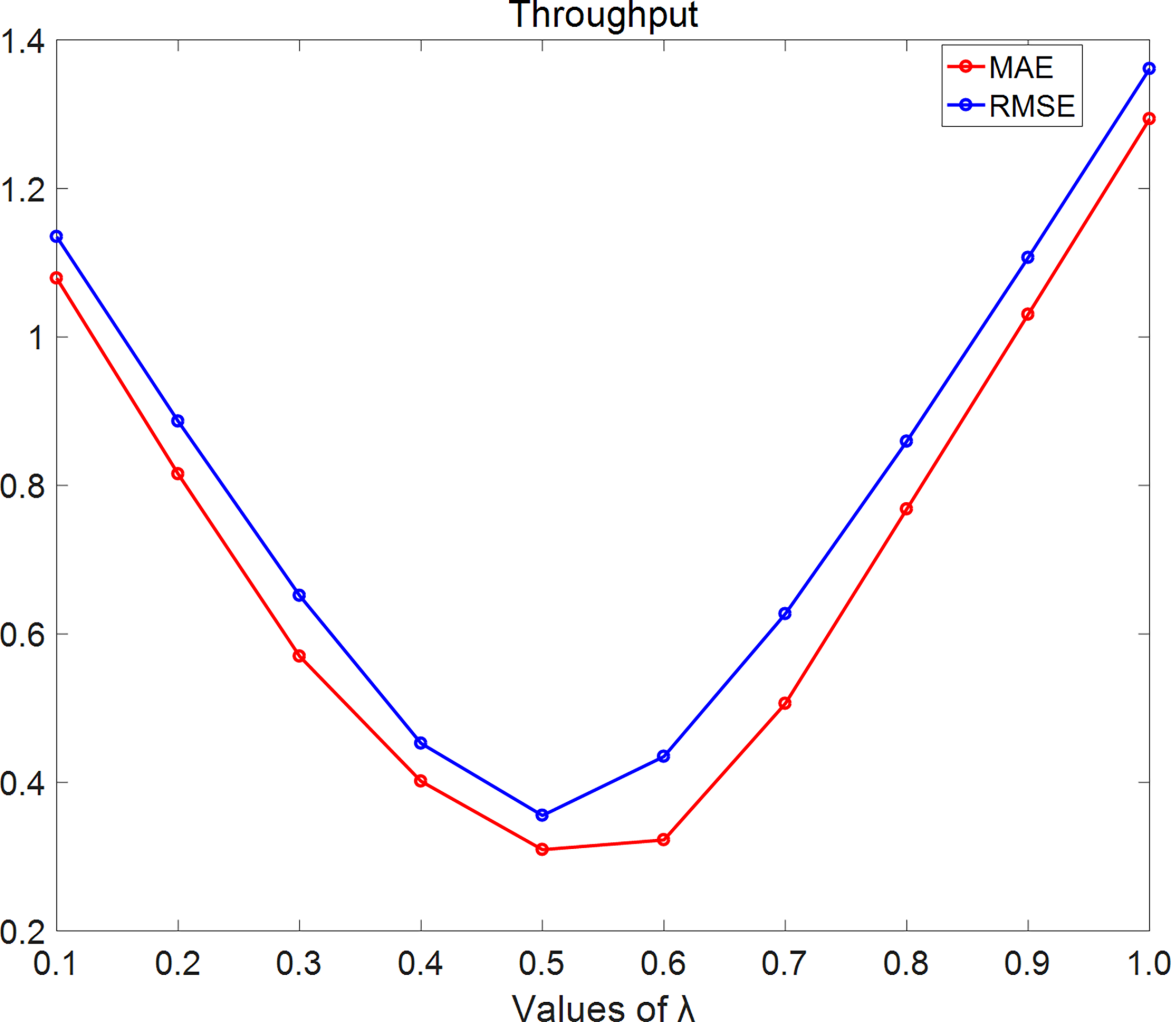
MAE changes with λ on throughput dataset. In this experiment, we set density = 10% and using throughput dataset. As the figure shows, MAE and RMSE firstly decrease with λ while λ<0.5 and then increase with λ while λ≥0.5.

In Fig 7, we set density = 10% and using throughput dataset. As the figure shows, MAE and RMSE firstly decrease with λ while λ< 0.5 and then increase with λ while λ≥0.5.

**Fig 8:**
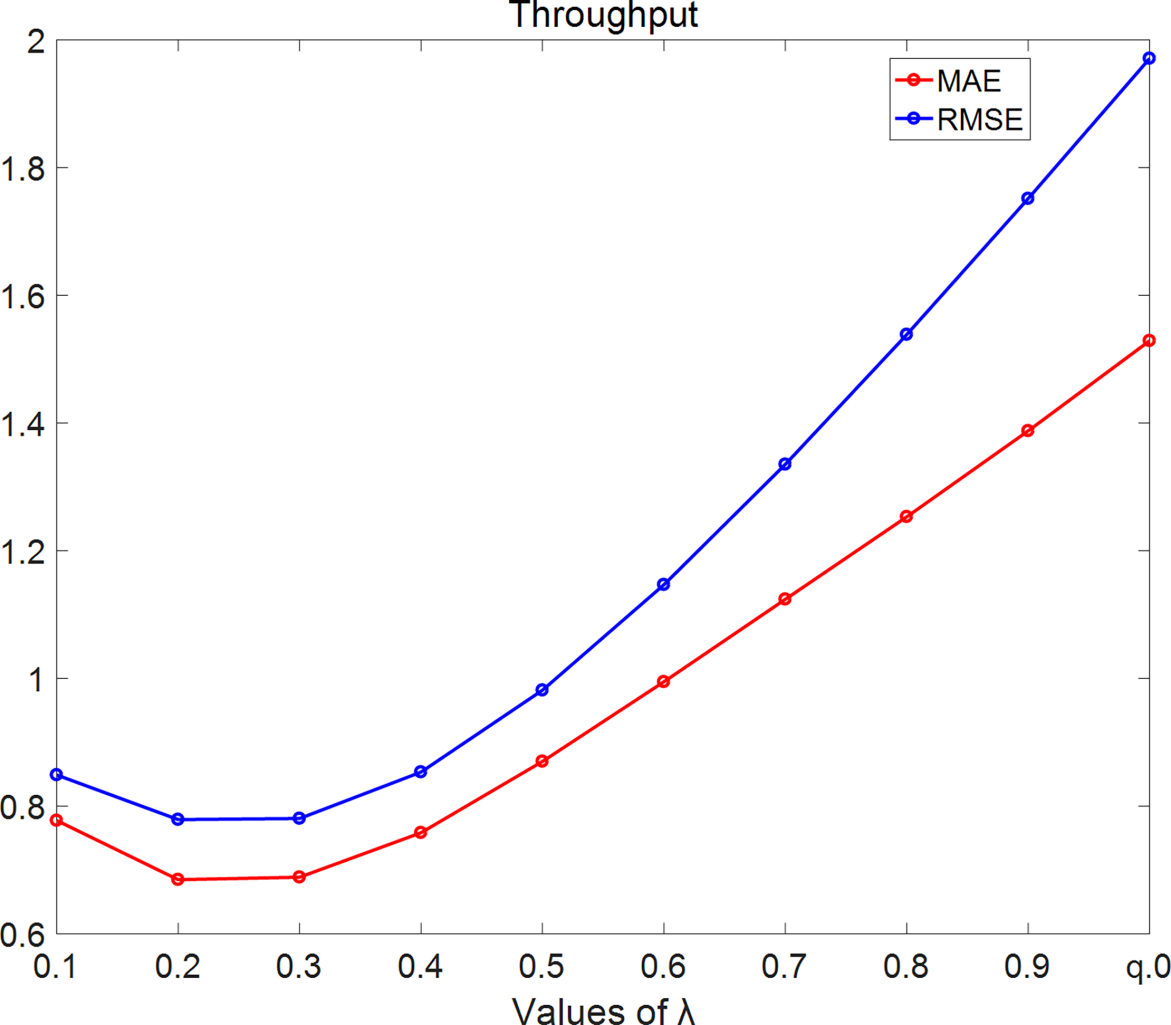
RMSE changes with λ on throughput dataset. In this experiment, we set density = 25% and using throughput dataset. As the figure shows, MAE and RMSE firstly decrease with λ while λ< 0.3 and then increase with λ while λ≥0.3.

In Fig 8, we set density = 25% and using throughput dataset. As the figure shows, MAE and RMSE firstly decrease with λ while λ<0.3 and then increase with λ while λ≥0.3.

From these experiments, we can see λ is a key factor affecting the values of MAE and RMSE. When given a matrix density, we can do an experiment to get the best λ to predict using HY method.

## Discussion

**Fig 9:**
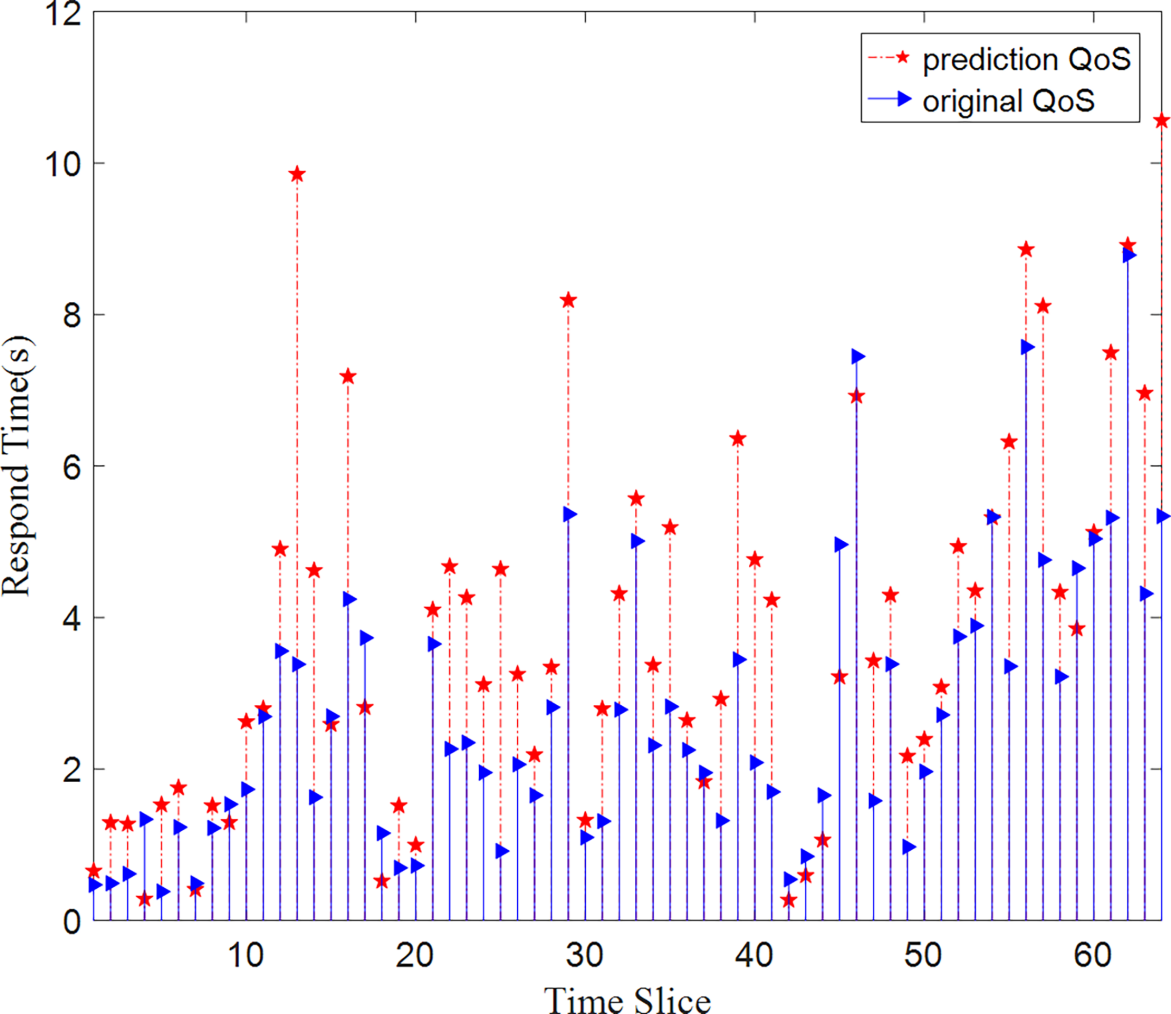
Prediction values VS original values on the respond-time dataset. The HY method has an effective prediction on QoS prediction. Compared to the original data, this method has an acceptable deviation on the respond-time dataset.

**Fig 10:**
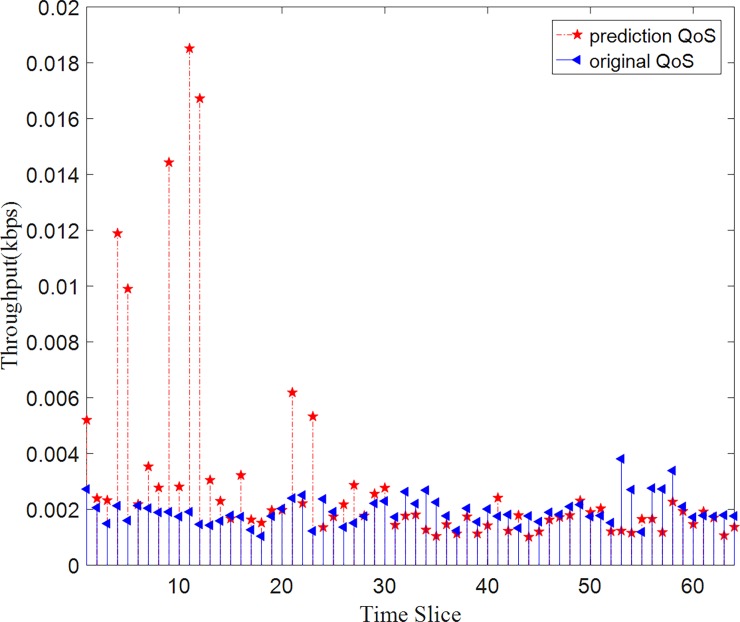
Prediction values VS original values on throughput dataset. The HY method has an effective prediction on QoS prediction. Compared to the original data, this method has an acceptable deviation on throughput dataset.

As we can see in Figs 9 and 10, The HY method has an effective prediction on QoS prediction. The method has an acceptable deviation on both dataset respond-time and throughput dataset. From the above experiments, we can analyze why these methods perform so differently with different parameter setting. The FFM-1 method makes a prediction based on similar users and FFM-2 method predicts based on similar services of the target object. From the analysis of dataset distribution, we can see it is a three-dimension matrix with 142 users, 4500 web services, and 64-time slices. Since the number of services is much more than the number of users, the similarity of users is more trusting than the similarity of services. So FFM-2 method has a higher prediction error than FFM-1 and HY methods. While we apply these methods to the real-world scenario, there will be far more than 142 users and far more than 4500 web services. They will perform better than in the laboratory experiments.

## Conclusions

This paper proposed a novel model to predict QoS values by extracting feature points and calculating the similarity of QoS temporal sequences. It's the first work to measure the similarity by DTW distance. The paper presented three prediction method: user-based prediction, service-based prediction, and hybrid prediction. Besides, this paper demonstrated the effectiveness of our model via a larger number of experiments with two datasets. Compared to the average methods, our proposed methods have better performance in the two datasets.

In the future, we will improve our work in the following two aspects. First, we will implement a framework for users to share the QoS experience and build a QoS database for the researchers. The small dataset limits the research and development of QoS prediction. Second, our proposed method only can predict the missing values based on the user-service-time matrix, we will extend our model to predict the near future QoS values based on the full matrix.
